# How Housing Conditions Determine the Welfare of Pigs

**DOI:** 10.3390/ani11123484

**Published:** 2021-12-07

**Authors:** Agnieszka Ludwiczak, Ewa Skrzypczak, Joanna Składanowska-Baryza, Marek Stanisz, Piotr Ślósarz, Przemysław Racewicz

**Affiliations:** 1 Department of Animal Breeding and Product Quality Assessment, Poznań University of Life Sciences, Słoneczna 1, 62-002 Suchy Las, Poland; ewa.skrzypczak@up.poznan.pl (E.S.); joanna.skladanowska-baryza@up.poznan.pl (J.S.-B.); marek.stanisz@up.poznan.pl (M.S.); piotr.slosarz@up.poznan.pl (P.Ś.); 2 Laboratory of Veterinary Public Health Protection, Poznań University of Life Sciences, Słoneczna 1, 62-002 Suchy Las, Poland; przemyslaw.racewicz@up.poznan.pl

**Keywords:** pigs, welfare, health, housing conditions, herd management

## Abstract

**Simple Summary:**

The increasing interest in the welfare of farmed animals has resulted in the development of alternative production systems for pigs. Access to outdoor pens or pastures is considered to improve welfare, as it is environmental-friendly and widely accepted by society. Outdoor housing allows pigs to freely display their natural behaviors, which is perhaps the most significant advantage of this type of housing vs. indoor housing systems. Among various indoor systems, bedding made of different materials, especially straw, appears to promote appropriate behavioral patterns in pigs, but it may also lead to respiratory disorders. However, the literature indicates that none of the described housing systems for pigs is perfect, and each one has some negative effects on welfare, management, and food safety.

**Abstract:**

Humans who care for pigs prefer an environment that not only allows the pigs to express their natural behaviors but also limits the development of aggression and stereotypes. Most of the behavioral and health problems encountered by pigs in barren, conventional conditions are solved by alternative housing systems. However, it is not known whether these systems are advantageous in terms of the performance of pigs. In this work, we review the effects of housing systems on pigs’ behaviors and performance, which are among the major indicators of the welfare of these animals. Research results point out that outdoor systems are more ideal for sows and fatteners than buildings. Nonetheless, outdoor housing is associated with two major effects in both groups: increased activity due to environmental exploration and higher space allowance, and increased incidence of injuries compared to indoor systems. Sows are more active when housed in groups, but they experience an increased frequency of injuries. According to the literature, group-housed sows give birth to healthy piglets with good daily weight gains. The difference in the fattening and slaughter performance of pigs raised indoors vs. outdoors remains unclear, and the results reported so far are inconsistent. Outdoor systems seem to be associated with a higher incidence of osteochondrosis and lesions of elbow and hock joints, whereas indoor systems cause a greater degree of body soiling in pigs. Based on the reviewed literature, it may be concluded that outdoor housing helps to solve behavioral issues in pigs but leads to other problems in pig production.

## 1. Introduction

The Five Freedoms identify the basic needs of animals [1]. Animals should not experience injuries or diseases, hunger or thirst, uncomfortable housing conditions, unsuitable environment, or stress. Although it is difficult to eliminate all these stressors from the production cycle of livestock, the European Union (EU) regulations suggest that the welfare of animals can be improved by increasing space allowance, facilitating social contact, and supporting the expression of explorative behaviors [2,3,4,5,6]. Each species has its own specific needs. According to the Council Directive, pigs should be raised in an environment that meets their needs and enable them to exercise and express their exploring behaviors. They should be housed in groups and provided favorable environmental conditions to prevent the development of aggressive behaviors as well as to promote natural behaviors, such as rooting. The enrichments used in pig farms should be food-like, chewable, and safe, and also provided in a sufficient amount (EU Directive 2008/120/EU) [7].

Although extensive outdoor housing piggeries are considered more ethical and welfare-friendly compared to intensive outdoor and indoor systems, from a practical perspective, outdoor rearing of pigs can lead to both management and welfare issues [8]. At present, a great part of the society opposes pig production under intensive farming conditions, not only because of decreased welfare but also due to negative environmental consequences. A significant advantage of extensive outdoor housing systems is that they allow the pigs to express their natural behaviors, which limits or even excludes the development of abnormal or aggressive behaviors [9,10]. However, outdoor systems also have some disadvantages. Pigs kept in pasture or outdoor pens are exposed to a number of uncontrolled and interacting environmental factors, including soil type and weather conditions, as well as the wild animals present in the farm area. Therefore, optimal farm location and proper management strategies are essential to ensure the high-level welfare of pigs, though there might be some difficult-to-predict interactions such as the ingestion of plants causing photosensitization [8]. Moreover, parasitism and piglet crushing are still frequent in extensive outdoor piggeries [10]. Outdoor pig farms are also associated with a higher risk of introduction and spread of African Swine Fever compared to indoor farms [11].

This review discusses the effects of housing conditions on the welfare of pigs. As behaviors, health (especially injuries, lameness, etc.), and performance or productive traits are believed to be primary indicators of welfare [12,13,14,15], the paper focuses on the influence of housing systems on these characteristics of sows, piglets, and fatteners. Among the production traits, the reproductive performance of sows, litter performance, fattening, and slaughter traits have been analyzed.

## 2. Behavioral Needs of Farmed Pigs

To ensure the high welfare of animals, it is important to understand their biological, physiological, and behavioral needs. Pigs are highly intelligent and social animals, the social status of which is determined by their age, body weight, and physical strength [16]. Among their natural behaviors, rooting seems to be very important and related to multiple roles. Pigs perform rooting in order to forage and to explore, while sows also root to build a nest prior to farrowing [17].

Pigs housed in barren environments often exhibit signs of boredom and frustration [18]. Environmental enrichments clearly improve the welfare of pigs by allowing them to express natural, species-specific behaviors and thus play a crucial role in the development of a welfare-friendly farm environment. On the other hand, the real effect of enrichments on pigs depends on many factors, including the type of enrichment, its adequate quantity, location, maintenance, and safety. Not all additives to barren farm environments are suitable enrichments for pigs [19]. Enrichments provided to pigs should be edible, chewable, safe, and frequently replaced or renewed so that the animals do not lose interest in them [20]. According to the Commission Recommendation (EU) 2016/336 [20], straw, green fodder, miscanthus, and root vegetables may be ‘optimal’ materials for pigs. When used as bedding, these materials promote rooting behavior, serve as a comfortable resting area, and absorb excreta [21]. Unfortunately, in light of African Swine Fever outbreaks in Europe, virus-infected straw, green forage, or hay have been identified as potential sources of the disease [22]. Furthermore, the use of natural enrichments, such as straw, is limited in some parts of the world due to higher production costs (including additional costs for straw and labor) compared to housing based on slatted floors. Moreover, housing systems with slatted floors may involve problems with slurry system management when substrates are used as environmental enrichments [23]. As suggested by Nannoni et al. [24], in no-bedding systems, pigs should be at least provided with hay or silage in racks placed above the floor. According to the Commission Recommendation (EU) 2016/336 [20], straw provided in racks, peanut shells, fresh wood, corn cobs, natural ropes, shredded paper, and pellets are ‘suboptimal’ enrichments for pigs. Though the deep-bedded systems based on straw appear to be welfare-friendly, they are not free from welfare and health problems. Studies analyzing the relationship between the use of straw as bedding and hygienic problems and development of pathogens have yielded contradictory results [23]. Moreover, pigs’ preference of floor type (deep-bedding vs. slatted floor) depends on the thermal conditions, as when temperatures are high, the animals will choose to lie on concrete floors to cool off [25]. With the exception of hot weather conditions, pigs prefer straw to concrete floors; however, substrates such as peat and compost are preferred by pigs over straw [23].

It has been demonstrated that the housing system determines the behavioral activity (e.g., time spent on rooting, lying down, inactive) of all groups of pigs. Piglets reared outdoor are more active compared to those housed on slatted floors [26]. Allowing piglets to express their play behavior improves their social skills and their ability to cope with adverse situations later in life [27]. Extensive outdoor systems enable pregnant sows to express nest-building and nursing behaviors [28]. All groups of pigs given access to outdoor runs exhibit wallowing, which is associated with multiple functions such as thermoregulation, protection against sunburn, elimination of skin parasites, and expression of social and sexual behaviors [29].

The majority of conventional husbandry systems do not address the behavioral needs of pigs [30]. Pigs exposed to barren, artificial environments and long-term stress may develop stereotypes and other behavioral abnormalities (e.g., sham chewing, bar biting, increased aggression, tail biting), which are considered as indicators of poor welfare [31,32,33]. Tail biting is commonly observed among pigs in commercial farms and is perhaps the most severe abnormal behavior. Outbreaks of this behavior have been observed at different phases of production and constitute a major problem for the pig industry due to the negative effects on the health, welfare, and performance of pigs, which lead to huge economic losses [34]. Excessive stocking density, competition for access to food and water, unfavorable or unstable temperature in buildings, insufficient ventilation, and high levels of noise, dust, and ammonia are some of the factors that trigger tail biting [35,36,37,38]. Pigs provided with access to straw display this behavior less frequently because they spend more time on rooting and other activities, and are less involved in manipulation on the tails of their pen-mates [39]. Although pigs reared in outdoor systems also exhibit aggression, the frequency and intensity of aggressive behaviors are lower compared to housing on slatted floors [38,40]. Extensive outdoor systems provide increased space allowance and a more diversified environment and thus limit or even prevent the development of stereotypies and aggressive or abnormal behaviors. Pig producers believe that tail biting can be overcome by tail docking. Although tail docking seems to decrease the incidence of tail biting, the procedure leads to the formation of neuromas in pigs’ tail tips associated with non-evoked pain and decreased nociceptive thresholds [41]. Neuromas are abnormal growths of nerve cells resulting from partial or total nerve damage [42]. It has been proven that tail biting can be limited or eliminated by providing environmental enrichments to pigs or by isolating the tail bitter [43]. The EU regulations do not allow for routine tail docking but suggest some measures to prevent the development of tail biting behavior [21], of which providing environmental enrichments is the most important. As the EU Directive from the year 2008 defined only general regulations [6], the European Commission has provided more detailed recommendations for preventing tail docking (EU 2016/336) [20]. These recommendations point to the use of the best type of materials as environmental enrichments to ensure that the basic behavioral needs of pigs are met.

## 3. Effects of Housing Conditions on the Welfare of Sows and Piglets

The housing system has been reported to strongly influence the maternal behaviors of sows. Freedom of movement promotes the expression of farrowing behaviors, such as nest-building [44]. It is also well known that environmental conditions determine the behavior of sows and piglets in the preweaning period and that an undesirable environment may increase the incidence of agonistic behaviors [45,46]. Prior to farrowing, sows exhibit nest-building behaviors such as foraging, rooting, and pawing [47]. If not provided with appropriate environmental conditions, they will redirect their nesting behaviors to head shaking, sham-chewing, drinker-playing, and drinking excessive amounts of water [48]. Such abnormal behaviors are mostly observed among sows that are raised under intensive housing conditions [49]. Furthermore, it has been proven that prepartum environmental stimuli (such as the presence of nesting material) promote nest-building behaviors in sows. Provision of a suitable substrate has a positive impact on the duration of nest-building behaviors and the amount of rooting observed prepartum [50]. Rosvold et al. [51] compared different nesting materials provided to sows before farrowing and observed that sows provided with straw and wood shavings expressed a higher number of total nest-building behaviors and nest-building elements (i.e., pawing, rooting, pushing, carrying and arranging material) compared to groups provided with peat and wood shavings and wood shavings only. Based on their results, the authors stated that both straw and peat promoted nest-building behaviors in comparison to no provision of these substrates.

Research data also underline that the housing system combined with suitable breed determines the maternal behavior in pigs. Free-range sows always check the bedding for the presence of piglets before laying down and move away from the piglets that are too close [52]. This behavior prevents piglet crushing, which is one of the major causes of mortality in litters [53,54]. Table 1 summarizes the effects of housing systems on sows’ health, behaviors, and performance based on the reviewed literature.

### 3.1. Behaviors and Health of Sows

Schrey et al. [55] analyzed the behaviors and health of sows kept in a novel group housing system. The housing system consisted of five single pens for farrowing with a common area in the center, a piglet area between two farrowing pens, and flexible iron bars and rubber bollards. In the 6-week study period, the sows spent significantly more time in the common area only in the fourth week, in comparison to pens. The authors observed that the behavioral activity of sows was influenced by the time of the day and flooring types. Suckling behavior was noted in sows in both pens and the common area, and cross-suckling was also observed. Based on their observations, the authors concluded that the novel housing system had a positive effect on the welfare of sows and piglets, as it promoted the expression of natural and social behaviors in pigs. Kim et al. [56] found that group-housed sows spent more time walking during gestation (*p* < 0.05) and eating during the farrowing period (*p* < 0.05) compared to those housed in individual gestation stalls. Sows housed individually were characterized by greater changes in backfat thickness (*p* < 0.05) and lower backfat thickness at weaning (*p* < 0.01), compared to group-housed pigs. Estienne et al. [57] also observed that the individual gestation housing system influenced the body condition of sows, including body weight (*p* < 0.01) and final body weight (*p* < 0.01). Contrary to Kim et al. [56], Estienne et al. [57] did not notice any effect of individual housing on the backfat thickness of sows. The authors observed that group-housed gilts experienced more severe injuries compared to gestation stall-housed gilts, but the level of recorded stereotypes was not affected by the housing system. Furthermore, the level of cortisol in serum collected on day 30 postmating did not vary among the examined groups of gilts. Several studies have shown that sows housed in groups may exhibit aggressive behaviors. Angermann et al. [58] compared two housing systems during gestation. The authors kept some sows in stable groups with a restrictive feeding regime and some in dynamic groups with group-adapted ad libitum feeding (Sow-Welfare-Optimized-Feeding, SWOF). They noted that the injury index was higher in the SWOF system compared to the stable-group system (0.74 vs. 0.54; *p* < 0.001). It was also observed that the injury index was dependent on the batch, time-point, and day of gestation. The results of the study revealed that the sow housing system did not influence the incidence of lameness among animals.

### 3.2. Sows’ Reproductive Performance

Kim et al. [56] observed that the feed intake and weaning-to-estrous interval of sows housed individually differed from that of group-housed sows (*p* < 0.05). The piglets of the group-housed sows had lower mortality, greater growth rates, and higher average daily gains compared to those of sows from individual gestation stalls (*p* < 0.05). In a study analyzing the reproductive performance of Złotnicka Spotted, a Polish native breed, Szulc [59] observed that sows kept outdoor were characterized by late first farrowing and a longer farrowing interval compared to conventionally housed pigs. These can be related to the effect of photoperiod on free-range sows, which leads to sexual maturity at an older age. The litter size at birth was greater in indoor-housed sows compared to the sows in the free-range system, although the number of piglets raised till the 21st day postpartum was the same in both groups. In another study, Szulc [60] compared Złotnicka Spotted sows placed under conventional housing conditions and those on an organic farm, outdoors. The author observed that sows kept under conventional housing conditions were characterized by a lower number of born-alive piglets, a higher number of weaned piglets, and lower piglet mortality compared to sows from the organic system. Literature data indicate that the mortality of piglets in outdoor systems is determined by environmental conditions (hypothermia, starvation) [61,62]. Luo et al. [63] examined the effects of a preweaning housing environment, postweaning housing conditions, and changes in postweaning housing conditions on the behaviors and postweaning performance of pigs. The authors observed that housing conditions influenced the behaviors of pigs at 3 weeks of age and at 47 days, just before the groups were switched between housing environments. They found that pigs reared in the enriched system spent more time exploring and chewing substrates, showed higher body weight gains, lower susceptibility to weaning stress, and better postweaning performance compared to those raised in a barren housing system.

## 4. Effect of On-Farm Environment on the Welfare of Growing Pigs and Fatteners

Housing pigs in indoor pens at high stocking densities can lead to health and behavioral problems [64,65]. Unlike conventional pig farms, organic farms focus on the well-being of animals as well as the environment, and therefore, meat production on organic farms is perceived as more ethical compared to commercial production [66]. Furthermore, pigs reared on organic farms are allowed to express species-specific behaviors, including the formation of social groups and social interactions, environment exploration, feeding through rooting, or wallowing in mud [67]. These animals are also more active and spend more time walking, playing, and laying, compared to pigs reared under intensive conditions [68]. Moreover, research indicates that harsh climatic conditions can affect the growth performance of pigs and suggest that crossbreeding can help overcome this issue [69,70]. The direction and significance of the effect of housing conditions on the growth and slaughter performance of pig fatteners are inconsistent. Some studies have reported that the housing system does not have an impact on most of the traits related to fattening performance and slaughter value [71]. On the other hand, some have concluded that the housing system influences only some characteristics of fattening performance [72], while some have pointed out the relationship between the housing system and performance of pig fatteners [24,73]. These inconsistent findings seem to indicate that there may be other factors interacting with the housing environment, mitigating the environmental impact on the performance of pigs.

### 4.1. Behaviors and Health of Weaner Pigs and Fatteners

Table 2 summarizes the literature data regarding the effects of housing conditions on the behaviors and health of weaner pigs and fatteners. Etterlin et al. [74] examined the effects of the housing system on the health of pig fatteners and found that free-range pigs more often suffered from osteochondrosis in elbows and hock joints compared to pigs kept indoors. The authors also noted that a higher percentage of free-range pigs had moderate and severe lesions on the elbow and hock joints compared to pigs raised under confined housing conditions. Liorančas et al. [75] observed that space allowance significantly influenced the behaviors of pig fatteners. Pigs kept at a stocking density of 0.5 m^2^/animal more frequently expressed biting and fighting behaviors compared to those kept at a stocking density of 1.2 m^2^/animal. Pigs that had greater space allowance spent more time on fixture exploration and resting compared to those kept in smaller pens. Street and Gonyou [76] also examined if space allowance affected the health and behaviors of pig fatteners. The authors did not observe any relationship between space and the incidence of lameness, flank and tail bites, or leg lesions. However, they noted that pigs kept in small, uncrowded groups spent the greatest amount of time on eating. The health and behaviors of pigs are determined not only by the housing conditions but also by the on-farm microclimate [77,78]. However, some studies have concluded that housing conditions and climate may not be the real cause of health complications in pigs. Done et al. [79] found that a dust concentration as high as 9.9 mg/m^3^ and ammonia concentration up to 37.0 ppm did not significantly affect the turbinate and lung scores of pigs, as well as the number of pathogenic bacteria isolated from the respiratory tracts of pigs. Scott et al. [80] compared the effects of a slatted floor system and straw-bedded housing on the health and behaviors of finishing pigs. The authors found that pigs reared on slatted floors had a higher incidence of lameness and bitten tails and suffered more severe bursitis compared to the pigs reared in a straw-bedded system. On the other hand, the presence of bedding also had an impact on pigs’ health, as fatteners reared on straw had more respiratory problems and a higher incidence of postweaning multisystemic wasting syndrome compared to pigs kept on a slatted floor. In addition, some differences in the behavioral activity were noted between the groups. Pigs reared on straw were more active compared to those reared on slatted floors, and the most frequent activity was straw manipulation. Similar to the above-cited works, the study by Temple et al. [81] showed that conventional housing systems were the major cause of bursitis in pigs farmed in pens on a slatted floor. Moreover, the authors found that the extent of body soiling was much greater among pigs kept under conventional and straw-bedded housing conditions compared to those in extensive housing systems in Spain and France. On the other hand, no effect of the housing system was found on the incidence of pigs’ behaviors such as huddling, shivering, and panting. According to Nannoni et al. [24], fatteners provided with more living space spent more time laying and less time in aimless exploration of slatted pen floor and more frequently expressed behaviors such as standing and walking compared to pigs provided with lower space allowance. Similar observations were made by Liorančas et al. [75], who noted that pigs reared in pens with extra space were more active and showed less aggression compared to those reared in limited space.

### 4.2. Fattening Performance of Pigs and Slaughter Traits

Table 3 summarizes the main findings regarding the effects of housing conditions on the fattening performance and slaughter traits of pigs. Patton et al. [82] compared the market performance of pig fatteners kept in a deep-bedded system containing hoop structures with that of pigs reared in a conventional housing system. The authors determined that the average daily gain of pigs kept under conventional conditions was greater compared to the group of pigs reared in the deep-bedded system. However, the conventional housing conditions were associated with lower feed efficiency. Although the carcass weight significantly differed between the studied groups, the slaughter performance as well as the loin eye area was similar. Juska et al. [72], in their study conducted in Lithuania, compared pigs reared indoors with those reared outdoors and found that outdoor-raised pigs were characterized by a higher daily gain in the growing and growing-finishing period, and a higher final weight in the growing and finishing period. Kozera et al. [71] compared fatteners from mating (Polish Large White × Polish Landrace) sows with (Duroc × Pietrain) boars kept indoors and outdoors with access to outdoor runs and noted that, under the Polish climatic conditions, the housing system did not affect the fattening performance and slaughter traits of those crossbreds. Acciaioli et al. [73] analyzed the influence of the housing system on the performance of Cinta Senese (CS), commercial Large White (LW), and their crossbreds (LW × CS) by assigning the animals to outdoor and indoor systems, under the climatic conditions of Italy. The authors observed that pigs reared indoors showed the best fattening performance: a slaughter weight of 140 kg was attained by LW pigs in 225 days, LW × CS pigs in 290 days, and CS pigs in 325 days. Pigs kept outdoors reached the same slaughter weight in a much longer period: LW × CS in 420 days and CS in 540 days. The lower body weight gains observed in outdoor-reared pigs were mainly due to discontinuity in feed access in the spring–summer period, while in autumn and winter the growth rates were higher due to access to acorns and chestnuts available in the woodland pastures, where the pigs were reared. Both Nannoni et al. [24] and Liorančas et al. [75] examined the effects of space allowance on pigs’ growth and fattening performance. The authors found a negative effect of lower space allowance on growth rate and body weight at slaughter. Similarly, Street and Gongou [76] examined the effects of stocking density on pigs’ fattening performance and observed that pigs reared in small groups, with a space allowance of 0.78 m^2^/animal, had a higher final body weight and greater average daily gains compared to those kept in large groups (0.52 m^2^). Škrlep et al. [83] studied the slaughter traits of fatteners reared under standard conditions and enriched conditions (greater space allowance with access to outdoor pens), and pigs switched to different pens during rearing. The authors observed that pigs kept in different pens during rearing were characterized by a lower live weight at slaughter and lower warm carcass weight compared to fatteners from other rearing systems. They also noted that mixed housing affected the backfat thickness of pigs. Based on their results, the authors concluded that the housing system did not influence most of the slaughter traits, including slaughter performance and muscularity traits.

## 5. Conclusions

Defining a single housing system that ensures high-level welfare of pigs, allows for ethical animal production, and assures food safety is difficult. According to the available data, outdoor systems and indoor systems with effective environmental enrichments have a positive influence on the behaviors of pigs. However, not all the alternative housing systems for pigs meet the expectations in terms of the high welfare of farmed animals. Moreover, the articles in this review are based on data gathered not only all over Europe but also in United Stated or Canada. Their comparability is limited, knowing that there is an interaction between the housing system and the local climatic conditions, and there are many more factors that can affect the performance of pigs (i.e., the breed, nutrition, season). Additionally, though outdoor housing systems help to overcome behavioral issues, they contribute to other problems in pig production and are the most influenced by local environmental conditions. Therefore, the implementation of outdoor hosing may differ between continents, countries and regions.

## Figures and Tables

**Table 1:** Effect of housing system on sows’ health, behaviors, and performance.

Authors	Environment and Housing Conditions	Affected Traits
Estienne et al. (2005)	Area: Virginia, USA Season: October, November, December Animals: Gilts at first gestation Factor: Individual gestation stalls vs. group housing (3 gilts/gestation pen) Gestation pens (3.1 × 1.7 m; 5.27 m^2^) partially slatted concrete floor; located in a mechanically ventilated building; mean high temperature was 22.4 °C and mean low temperature was 17.6 °C. Gestation stalls (0.6 × 2.0 m; 1.2 m^2^) with partially slatted concrete floor, located in a curtain-sided building; mean high temperature was 19.8 °C and mean low temperature was 15.8 °C.	Effect: Pens vs. stalls; final body weight 170.6 vs. 166.3 kg, *p* < 0.01; change in body weight 11.0 vs. 6.7 kg, *p* < 0.01; lesions score greater in stalls No effect on: backfat thickness, lameness score; display of stereotypies
Szulc (2011)	Area: Poland Season: - Animals: Złotnicka Spotted, a Polish native breed Factor: Outdoor vs. indoors on shallow bedding	Effect: Outdoor pens compared to indoors: later age of first farrowing, longer farrowing interval, lower litter size at birth No effect on: number of piglets raised till 21st day postpartum was similar
Szulc (2012)	Area: Poland Season: - Animals: Złotnicka Spotted, a Polish native breed Factor: Conventional housing conditions vs. organic outdoor farm	Effect: Organic vs. conventional; number of piglets born alive 9.42 vs. 8.87, *p* < 01; mortality of piglets 16.03% vs. 9.96%, *p* < 0.01 No effect on: age at first farrowing, intervals between litters, number of piglets reared till day 21 postpartum
Kim et al. (2016)	Area: Republic of Korea Season: - Animals: crossbred sows (Landrace×Yorkshire) in their 3–4 parities Factor: Individual gestation stalls vs. group housing Gestation stalls (2.20 × 0.65 m) with fully slatted concrete flooring. Group-housed sows were kept in pens (10.4 × 5.4 m), 16 sows/pen. All sows were moved to farrowing crates (2.2 × 0.65 m) on day 109 of gestation.	Effect: Gestation stalls compared to group housing; lower backfat thickness at 1 day of lactation (*p* = 0.03); smaller backfat thickness change in 1–21 days of lactation (*p* = 0.04); lower feed intake (*p* = 0.04), shorter weaning-to-estrous interval (*p* = 0.04); lower number of weaned piglets (*p* = 0.03); lower growth rates (kg/d) in piglets (*p* = 0.01); lower average daily gain (*p* = 0.04); less time walking during gestation (*p* = 0.01); less time eating during the farrowing period (*p* = 0.03) No effect on: Number total born and born-alive piglets, birthweights of piglets; time spent on: ventral laying, sitting, standing, and drinking during gestation
Angermann et al. (2021)	Area: Brandenburg, Germany Season: January and June 2018 Animals: Danish genetic Factor: Two housing systems during gestation, existing system based on stable groups with restrictive feeding regime vs. dynamic groups with Sow-Welfare-Optimized-Feeding (SWOF) Existing system, sows were kept in a stable group (average 48 pigs) in a pen (7.70 × 17.50 m) divided by the trough in the middle into two groups of 18–25 sows (pen size of 3.63 × 17.50 m per group); no functional area; fully slatted floor; negative pressure ventilation. SWOF system, sows were kept in large dynamic groups (average 105 sows); partially slatted floor; functional areas (activity and lying area, ad libitum liquid feeding areas; negative pressure ventilation.	Effect: Stable groups compared to SWOF system; lower injury index No effect on: lameness; litter birthweight; number of born piglets; piglets born alive; stillborn; mummified piglets
Luo et al. (2020)	Area: Wageningen, the Netherlands Season: - Animals: Pigs (Tempo × Topigs 20) Factor: Barren housing system vs. enriched; part of pigs switched between systems at 47 days of age Barren system 8.6 m^2^ pens; solid floor and a small slatted area; toys: Enriched system; 17.1 m^2^ pens; the enriched part contained 1.7 kg of straw, 300 L of sawdust, and 270 L of peat as substrates on the floor; toys.	Effect of barren system: - Behaviors at 3 weeks of age: less time spent on exploring, more pen-directed exploring, less chewing, more pen-directed chewing, aggression, more manipulation; - Behaviors at 47 days of age: more time inactive, less exploring, more pen-directed exploring, less chewing, more pen-directed chewing, more manipulation and mounting; Effect of enriched system: - Behaviors of pigs at 3 weeks of age: less time spent on exploring, more pen-directed exploring, less chewing, more pen-directed chewing, aggression, more manipulation; - Behaviors at 47 days of age: more time inactive, less exploring, more pen-directed exploring, less chewing, more pen-directed chewing, more manipulation and mounting; Effect of housing system: - before and after the switch on body weight gains on days 109–130; - after the switch on body weight gains on days 46–130. No effect on: inactivity, social behaviors and mounting at 3 weeks of age; play and aggression at 47 days of age.

**Table 2:** Effect of housing conditions on the behaviors and health of weaner pigs and fatteners.

Authors	Farm Environment	Affected Traits
Nannoni et al. (2019)	Area: Italy Season: - Breed: Crossbred Duroc × (Landrace × Large White) barrows Factor: space allowance: 1- or 1.3-m^2^ space/head; 5 animals per pen; fully slatted floor; temperature-and humidity-control (22–24 °C, 70–80%); pigs slaughtered at 160 kg body weight.	Effect: Space allowance of 1.3-m^2^ space/head leads to more time spent on lateral and total recumbency; less time spent on exploration of the pen floor, more time spent on drinking, walking, and standing compared to 1-m^2^ space/head. No effect on: Sitting inactive, sternal recumbency, eating, social interactions.
Etterlin et al. (2016)	Area: Sweden Season: - Animals: crossbred Hampshire (Yorkshire×Landrace) Factor: Confined indoor system vs. free-range Confined indoor housing, 5–7 pigs/pen (12 m^2^); solid concrete floors, minimal bedding in the resting area, slatted concrete floor in the defecation area. Free-range: 50 pigs/pen (a 90-m^2^ indoor area: feeding area with a solid concrete floor, a resting area with deep straw bedding, a defecation area with a slatted concrete floor; outdoor area consisted of a run with a concrete floor (26 m^2^) and access to pasture (approximately 2500 m^2^)	Effect: Free-range compared to confined indoor housing; osteochondrosis in elbows 69% vs. 50%; *p* < 0.05; osteochondrosis in hock joints 33% vs. 16%; *p* < 0.05; more severe OC ^a^ in the humeral condyle than confined pigs (*p* < 0.05); higher severity (*p* < 0.001) of OC in the hock joints.
Liorančas et al. (2006)	Area: Lithuania; Season: - Animals: crossbred females and castrated males; Danish Landrace × Danish Yorkshire × Danish Duroc Factor: 0.5 vs. 1.2 m^2^ space/pig, indoor housing system	Effect: 0.5 vs. 1.2-m^2^ space/pig Time spent on biting 8.2% vs. 1.8% Time fighting 6.2% vs. 0.5% Time of fixture exploration 19.8% vs. 28.1% Time spent on resting 21.7% vs. 25.3%
Street and Gonyou (2008)	Area: Canada Season: - Animals: - Factor: small crowded conditions (18 pigs at 0.52 m^2^/head), small uncrowded conditions (18 pigs at 0.78 m^2^/head), large crowded (108 pigs at 0.52 m^2^/head), large uncrowded (108 pigs at 0.78 m^2^/head). Pen dimensions: 5.8 × 1.6 m, 5.8 × 2.4 m, 9.8 × 5.8 m, and 14.6 × 5.8 m for small crowded, small uncrowded, large crowded, and large uncrowded, fully slatted floors.	Effect: Space allowance affected time spent on eating (*p* = 0.003); Group size affected: time sitting (*p* = 0.003; more in large groups); time lying ventrally (*p* = 0.002; more in small groups); time lying laterally (*p* = 0.012; more in small groups) No effect on: lameness, flank bites, leg lesions, tail bites, removal because of injuries
Scott et al. (2006)	Area: United Kingdom Season: April 2002 to December 2004 Animals: (Large White × Landrace) × Large White pigs Factor: Fully slatted vs. straw-bedded housing system; Straw-bedded pens measured 5.8 × 3.7 m; fully-slatted pens measured 5.5 × 3.7 m, each pen was provided with a hanging toy.	Effect: higher incidence of lameness (*p* < 0.001) and bitten tails (*p* < 0.001) and more severe bursitis (*p* < 0.001) in pigs on slatted-floors compared to straw-bedded system; more respiratory problems (*p* < 0.01) and a higher incidence of postweaning multisystemic wasting syndrome (*p* < 0.01) among animals in a straw-bedded system compared to slatted-floors; more activeness (*p* < 0.001) of pigs in a straw-bedded system compared to those reared on slatted floors No effect on: lesion score
Temple et al. (2012)	Area: Spain, France Season: - Animals: Iberian pigs, Mallorca Black pigs Factor: 5 production systems: conventional, straw-bedded, intensive Iberian, extensive Iberian, extensive Mallorca Black pig; Conventional system; pigs kept on concrete floors (64% on fully slatted floor, 36% on partly slatted floor without bedding); space allowance ranged from 0.20 to 1.56 m^2^/head Straw-bedded system, space allowance ranged from 0.3 to 3.0 m^2^/head; Intensive Iberian, housed on slatted floors (18% on partly slatted floors without bedding, 47% on concrete floor with either a resting area on straw or an outdoor access and 19% housed in outdoor paddocks on deep sand or straw), space allowance ranged from 0.30 to 5.4 m^2^/head; Extensive Iberian, housed on paddocks; average space allowance was 430 m^2^/head. Extensive Mallorcan Black, housed on paddocks; average space allowance was 692 m^2^/head.	Effect on: body condition, bursitis, soiled body surface No effect on: huddling, shivering, panting

^a^ OC—osteochondrosis.

**Table 3:** Effect of housing conditions on the fattening performance of pigs and slaughter traits.

Authors	Research Details	Affected Traits
Patton et al. (2008)	Area: Castana and Ames; USA Season: - Breed: - Factor: Conventional housing vs. deep-bedded system with hoop structures; 0.70-m^2^ space/head.	Effect: Conventional vs. deep-bedded system with hoop structures; average daily gain (kg/day) 1.07 vs. 0.81, *p* < 0.01; feed efficiency 0.42 vs. 0.52, *p* < 0.01; shrink (%) 4.48 vs. 2.32, *p* < 0.01; carcass weight (kg) 82.75 vs. 79.15, *p* < 0.05; backfat thickness (mm) 15.24 vs. 13.72, *p* < 0.01. No effect on: Slaughter performance, loin area
Acciaioli et al. (2002)	Area: Italy Season: - Animals: Cinta Senese; Large White; Large White×Cinta Sense; castrated males and females; Factor: Indoor vs. outdoor housing (woodland pastures with oaks and chestnuts)	Effect: Lower fattening performance and weight gain of pigs reared outdoors compared to indoor-reared animals.
Nannoni et al. (2019)	Described in Table 2.	Effect: 1 or 1.3 m^2^ space/head; Final body weight (day 224) 154.4 vs. 162.2, *p* = 0.02; Average daily gain (ADG) 140–224 days (kg/day) 0.631 vs. 0.686, *p* < 0.01; Overall ADG (kg/day) 0.583 vs. 0.619, *p* = 0.01; Gain-to-feed ratio 140–224 days, 0.206 vs. 0.223, *p* = 0.03; Overall gain-to-feed ratio, 0.256 vs. 0.271, *p* = 0.02. Space allowance of 1.3-m^2^ space/head leads to more time spent on lateral and total recumbency; less time spent on exploration of the pen floor, more time spent on drinking, walking, and standing compared to 1-m^2^ space/head. No effect on: carcass traits
Liorančas et al. (2006)	Described in Table 2.	Effect on: time spent on activities, body weight at slaughter, growth rate, slaughter performance No effect on: carcass weight, lean meat content
Juska et al. (2013)	Area: Lithuania Animals: Lithuanian White × Swedish Yorkshire × English Large Factor: Indoor vs. outdoor housing system; indoor in pens on straw-littered concrete floors; 18.5-m^2^ space/13 pigs; outdoor enclosures of 850 m^2^ area with three-wall shades; 13 pigs/enclosure.	Effect on: growth rate in the growing and growing-finishing period, final live weight in the finishing period, protein content in pork No effect on: initial and final weight in the growing period, growth rate in the finishing period, slaughter traits
Kozera et al. (2016)	Area: Poland; Season: from May to August (summer) Animals: Pigs from mating (Polish Large White × Polish Landrace) sows with (Duroc × Pietrain) boars. Factor: Indoor pens vs. indoor pens with access to outdoor runs Pigs of all experimental groups were kept in pens (3 × 3 m without litter. 5 pigs/pen) Group 1: fed a complete diet; kept indoors with free access to outdoor runs; Group 2 was fed a complete diet; was kept indoors; Group 3 was fed a complete diet with an increased ME ^a^ content; kept indoors with free access to outdoor runs; Group 4 was fed a complete diet plus green alfalfa forage; kept indoors with free access to outdoor runs; Group 5 was fed a complete diet plus green alfalfa forage; kept indoors; Group 6 was fed a complete diet with increased ME ^a^ content, plus green alfalfa forage; kept indoors with free access to outdoor runs.	Effect on: daily feed intake No effect on: fattening performance, slaughter traits
Škrlep et al. (2020)	Area: Germany Animals: crossbred pigs German Landrace × Pietrain; Factor: Standard conditions vs. enriched housing conditions (twice more space as under standard conditions and access to outdoor pens) vs. mixed housing (two pens of mixed during rearing).	Effect on: live body weight, warm carcass weight, measurement of backfat thickness: at withers, at second-to-third last rib, fat corresponding to loin eye area No effect on: Slaughter performance, carcass length, muscularity traits
Street and Gongou (2008)	Described in Table 1.	Effect on: final body weight, average daily gain No effect on: initial body weight

^a^ ME—metabolizable energy.

## Data Availability

No new data were created or analyzed in this study. Data sharing is not applicable to this article.
